# An Update of Long-Noncoding RNAs in Acute Kidney Injury

**DOI:** 10.3389/fphys.2022.849403

**Published:** 2022-03-08

**Authors:** Lina Yang, Bo Wang, Liang Ma, Ping Fu

**Affiliations:** Kidney Research Institute, Division of Nephrology, West China Hospital of Sichuan University, Chengdu, China

**Keywords:** acute kidney injury, long non-coding RNA, sepsis, ischemia–reperfusion, post-contrast AKI, kidney transplant, drug induced AKI

## Abstract

Acute kidney injury (AKI) is a global public health concern with high morbidity, mortality, and medical costs. Despite advances in medicine, effective therapeutic regimens for AKI remain limited. Long non-coding RNAs (lncRNAs) are a subtype of non-coding RNAs, which longer than 200 nucleotides and perform extremely diverse functions in biological processes. Recently, lncRNAs have emerged as promising biomarkers and key mediators to AKI. Meanwhile, existing research reveals that the aberrant expression of lncRNAs has been linked to major pathological processes in AKI, including the inflammatory response, cell proliferation, and apoptosis, *via* forming the lncRNA/microRNA/target gene regulatory axis. Following a comprehensive and systematic search of the available literature, 87 relevant papers spanning the years 2005 to 2021 were identified. This review aims to provide and update an overview of lncRNAs in AKI, and further shed light on their potential utility as AKI biomarkers and therapeutic targets.

## Introduction

Acute kidney injury (AKI) is a severe clinical syndrome characterized by an abrupt decrease in kidney function, as measured by high blood creatinine and decreased urine volume within 7 days (Kellum et al., 2012). Epidemiologic studies have found that about 13.3 million people worldwide suffer from AKI each year (Mehta et al., 2015). Furthermore, the global health burden of AKI-related mortality is far beyond that of breast cancer, heart failure, or diabetes (Lewington et al., 2013). Therefore, finding effective prevention and treatment approaches are indispensable. Clinically, the cause of AKI can be divided into three categories: pre-renal (impaired renal perfusion), intra-renal, and post-renal (urinary tract obstruct). Among these, sepsis, and ischemia/reperfusion (I/R) are the common leading causes of AKI. Besides, post-contrast AKI (CI-AKI), transplant-related AKI, and drug or toxin induced AKI have become prevalent and important as medical care evolves as well. The pathogenesis of AKI is multifactorial and has been overwhelmingly implicated in the etiology of AKI (Kinsey and Okusa, 2011).

Even though our understanding of the pathophysiology of AKI is continually expanding, the precise processes remain unknown. Following the improvements and cost reductions of high throughput RNA-sequencing, considerable research has reported that the non-coding RNAs (ncRNAs), especially long non-coding RNAs (lncRNAs), are strongly associated with the pathophysiological process underlying AKI. As the quantity of research on lncRNAs has grown fast, in order to offer a thorough and up-to-date overview of the function of lncRNAs in AKI, we briefly introduce the lncRNA and systematically review the mechanism of functional lncRNAs in AKI due to various etiologies. Finally, we highlight certain aspects of the lncRNA mechanism in AKI, as well as future research directions.

## Overview of lncRNAs

LncRNAs are a subtype of ncRNA that longer than 200 nt with no protein-coding potential and make up the most considerable portion of the mammalian non-coding transcriptome (Mercer et al., 2009; Derrien et al., 2012). Sequence analysis of lncRNAs between different species revealed that lncRNAs presented the characteristics of low DNA sequence conservation and low expression. Commonly, according to lncRNA location concerning protein-coding genes, lncRNAs could be classified as either intergenic or intragenic. Intergenic lncRNAs do not intersect with any protein-coding genes. In contrast, intragenic lncRNAs overlap with protein-coding genes and could be further sorted into antisense, bidirectional, intronic, and overlapping sense lncRNAs. In parallel, lncRNAs could also be categorized based on their length, location, transcript properties, regulatory mechanisms, function, and so on (Statello et al., 2021).

Most lncRNA species are transcribed by Pol II. It also could be capped, spliced, and polyadenylated, exported like mRNA. However, after being transcribed by Pol II, lncRNAs will further fold into the complex secondary or tertiary structures. Those high-ordered structures showed higher conserved than their primary sequence. Diversified structure enables lncRNAs to participate in life activities and disease progress by interacting with DNA, RNA, or protein to regulate gene expression at multiple levels (epigenetic regulation, transcription regulation, and post-transcriptional regulation). Among these, research on the roles of lncRNAs in post-transcriptional regulation has been investigated the most. Three major mechanisms of lncRNA regulate the gene expression in post-transcriptional: a. binding to RNA or structures to form a lncRNA-protein complexes directly; b. pairing with the other RNAs to recruit proteins; c. competing endogenous RNAs or ‘sponges’ of miRNAs to restrict miRNA availability to target mRNAs (Marchese et al., 2017; Salviano-Silva et al., 2018). Aside from the structure, the location of lncRNAs in cells also exerts an intensive effect on their functions (Chen, 2016). In addition to being located in the nucleus, lncRNAs have been detected in additional subcellular sites such as mitochondria and exosomes (Bridges et al., 2021). Moreover, these organelle-specific lncRNAs are intensively implicated in organelle homeostasis, which is always plays a role in the occurrence and development of the disease (Li et al., 2021b).

Currently, research methodologies for investigating the functions of lncRNAs in disease include lncRNA identification, lncRNA characteristic analysis, functional investigations, and molecular processes (Gao et al., 2020a). Similarly, advances in experimental equipment and computational methodologies in lncRNAs study have occurred in recent years. Among these advancements, fully functional sequencing databases stands out. First, with the introduction of third generation sequencing technology and lncRNAs databases, lncRNA screening has grown completer and more reliable. Second, emerging bioinformatics algorithm, database RACE, and fluorescence *in situ* hybridization (FISH) offer great assistance in annotating the characteristic of lncRNAs, such as coding potential, location information on the genome, secondary structure, correlation with the diseases, full-length analysis, and cell localization (Liu et al., 2018). Then, the roles of lncRNAs are generally validated by overexpression or Knock down of specific lncRNAs, and their role in diseases development is studied by assessing changes in biological behaviors *in vitro* and *in vivo* tests. Finally, by combining the prediction work of databases and experiment technology such as DNA-FISH (Gelali et al., 2019), chromatin immunoprecipitation (Nakato and Sakata, 2021), RNA-pull down, luciferase reporters (Cao et al., 2019), and methylation-specific PCR (Hernández et al., 2013), the specific molecular regulation network between the protein, RNA, or DNA, and the lncRNAs could be illustrated.

Based on the strategies and technologies mentioned above, studies have identified that lncRNAs serve a crucial role in various biological processes, ranging from the cell cycle, growth, apoptosis, and immune responses to some tissue-specific physiology. Many lncRNAs have been verified to have links with prevalent diseases, such as cancer, neurological and cardiovascular diseases (Fenoglio et al., 2013; Peng et al., 2017; Fernandes et al., 2019). Simultaneously, increased emphasis has been placed on the significance of lncRNAs in renal disorders.

## LncRNAs in Septic AKI

### LncRNAs Globally Changed in Septic AKI

**Figure 1:**
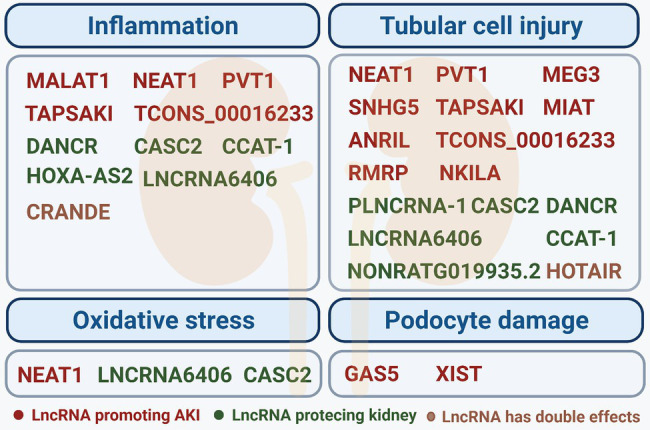
Summary of pathologic processes of long-noncoding RNAs (lncRNAs) in septic acute kidney injury (AKI). To summarize our findings, lncRNAs that primarily participate in four pathologic processes, which include inflammation, tubular cell injury, oxidative stress, and podocyte damage in septic AKI, are classified as indicated. In each category, the lncRNAs found to act as a promoting factor in the sepsis AKI are highlighted in red, while the lncRNAs found to act as protective factors are depicted in green, and the lncRNA with a dual effect are depicted in brown.

Patients who suffer from sepsis are associated with a higher risk of incidence and mortality of AKI. The pathogenesis of sepsis-induced AKI is very complex and remains unclear. Mounting evidence suggests that lncRNAs could exhibit diverse functions in physiological and pathological processes of septic AKI (Figure 1, Table 1). The large-scale analyses showed that a significant expression difference of lncRNAs between AKI and normal control does exist, both in mice and humans. Chun-Mei and collaborators identified 5,361 up-regulated lncRNAs and 5,928 down-regulated lncRNAs differentially expressed in septic AKI humans compared with a control group (Chun-Mei et al., 2016). Another meta-analysis summarized the 38 independent studies and recognized 31 remarkably dysregulated lncRNAs in total, some of which have been considered the potential predictive biomarkers and therapeutic targets of AKI and further verified in the research as follows already (Ma et al., 2021).

**Table 1:** LncRNAs in septic AKI.

AKI	lncRNA	Expression	Regulation network	Function	Reference
Septic AKI	MALAT1	up	miR-146a/NF-κB	Promote cytokine creation and immune response	Ding et al. (2018)
	NEAT1	up	miR-22-3p/NF-κB	Promote cell apoptosis	Feng et al. (2020)
			let-7b-5p/TRAF6	Promote inflammation	Gao et al. (2020b)
			miR-93-5p/TXNIP	Promote apoptosis, inflammation, and oxidative stress	Yang et al. (2021b)
			miR-27a-3p/TAB3	Promote cell apoptosis	Wang et al. (2020a)
			miR-125a-5P/TRAF6/TAK1	Modulate macrophage polarization	Wang and Guo (2020)
	PVT1	up	miR-17-5p/ NF-κB	Promote cell apoptosis	Yuan et al. (2021b)
			miR-20a-5p/NLRP3	Promote cell pyroptosis and inflammatory factors creation	Deng et al. (2021b)
	DLX6-AS1	up	miR-223-3p/NLRP3	Improve cytotoxicity	Tan et al. (2020)
	MEG3	up	miR-21/PDCD4	Promote cell apoptosis	Yang et al. (2018)
			miR-18a-3p/GSDMD	Promote pyroptosis	Deng et al. (2021a)
	TapSAKI	up	miR-22/PTEN/TLR4/NF-κB	Promote cell apoptosis and inflammation response	Shen et al. (2019)
			miR-205/IRF3	Enhance cytotoxicity	Han et al. (2021b)
	SNHG14	up	miR-93/IRAK4/NF-κB	Accelerate cellular injury	Shi et al. (2021)
			miR-495-3p/HIPK1	Inhibit cell proliferation and autophagy	Yang et al. (2021a)
	SNHG5	up	miR-374a-3p/TLR4/NF-κB	Promote cell apoptosis	Wang et al. (2021)
	MIAT	up	miR-29a	Promote cell apoptosis	Zhang et al. (2019)
	ANRIL	up	miR-199a/TLR4/NF-kB	Promote cell apoptosis	Zhu et al. (2020b)
	TCONS_00016233	up	miR-22-3p/AIFM1	Promote LPS-induced HK-2 cell apoptosis and the expression of IL-1b and TNF-a	Zhang et al. (2020b)
	SIKIAT1	up	miR-96-3p	Promote cell apoptosis	Lu et al. (2020b)
	RMRP	up	miR-206/DPX5	Promote cell apoptosis	Zhang et al. (2021)
	NKILA	up	miR-140-5p/CLDMA	Promote cell apoptosis	Han et al. (2021a)
	XIST	up	miR-15a-5p/ CUL3	Enhance podocyte cell apoptosis	Xu et al. (2019)
	GAS5	down	PI3K/AKT	Promotes podocyte injury	Fang et al. (2018)
	PlncRNA-1	down	-	Promote cell proliferation, inhibit apoptosis and autophagy	Fu et al. (2018)
	DANCR	down	miR-214/KLF6	Suppress cell apoptosis and cytokine creation	Zhao et al. (2020)
	lncRNA6406	down	miR-687/PTEN	Alleviate inflammation, oxidative stress, and inhibit apoptosis	Liu et al. (2020)
	HOXA-AS2	down	miR-106b-5p/Wnt/β-catenin/ NF-κB	Inhibit the inflammation	Wu et al. (2020b)
	LINC00261	down	miR-654-5p/SOCS3/NF-κB	Improve cell viability, suppress the apoptosis, and reduced the generation of inflammation cytokines	Li et al. (2021c)
	CASC2	down	miR-155/NF-κB	Inhibit inflammation factors creation, cell apoptosis and oxidative stress	Wang et al. (2020d)
			miR-545-3p/PPARA	Facilities cell viability and restrain cell apoptosis migration EMT and oxidative stress	Hu et al. (2021)
	CCAT-1	down	miR-155/SIRT1	Attenuate inflammatory response and apoptosis	Lu et al. (2020a)
	NONRATG019935.2	down	P53	Suppress the apoptosis	Ding et al. (2021)
	CRNDE	up	TLR3/NF-κB	Promote kidney injury	Sun et al. (2019)
			miR-146a /TLR4/NF-κB	Accelerate LPS-induced inflammation and apoptosis	Wu et al. (2020a)
		down	miR-181a-5p	Accelerate LPS-induced inflammation and apoptosis	Wang et al. (2020c)
	HOTAIR	up	miR-34a/Bcl-2	Inhibit the apoptosis of kidney tissues	Jiang et al. (2019b)
			miR-22/HMGB1	Promote HK-2 cell apoptosis	Shen et al. (2018)

### LncRNAs Promoting Septic AKI

LncRNAs that are up-regulated in septic AKI are always a focus for investigation. As mentioned before, lncRNAs can act as competing endogenous RNAs to compete with mRNAs for binding to the same miRNAs and influence the expression of miRNA-targeted transcripts. The interplay between the lncRNAs, miRNAs, and mRNAs is called a ceRNA crosstalk. Constructing ceRNA regulatory networks is the most common method for studying the biological functions of lncRNAs (Lan et al., 2019). LncRNA metastasis-associated lung adenocarcinoma transcript 1 (MALAT1) is one of the well-studied lncRNA involved in septic AKI. The expression of MALAT1 is increased in sepsis patients’ serum, experimental animal kidney tissue, and cell lines. MALAT1 could promote kidney injury *via* downregulating miR-146a and activating the nuclear factor-κB (NF -κB) in LPS induced AKI. Correspondingly, silencing MALAT1 displayed a significant renal protective effect (Ding et al., 2018). Some medications, such as paclitaxel and dexmedetomidine, could also exert their therapeutic effect by reducing MALAT1 expression in tubule cells (Xu et al., 2020; Zhu and Lu, 2020a).

Numerous scholars have also intensively studied the function of long noncoding RNA nuclear-enriched abundant transcript 1 (NEAT1) in sepsis-induced AKI. Depletion of NEAT1 could attenuate sepsis-induced AKI *via* regulating the miR-22-3p/NF-κB pathway (Feng et al., 2020). Furthermore, lower expression of NEAT1 may obstruct the development of LPS-induced injury and inflammation in HK-2 cells by targeting the let-7b-5p/TRAF6 axis or miR-93-5p/TXNIP axis (Gao et al., 2020b; Yang et al., 2021b). siNEAT1 could alleviate the cecal ligation punctures (CLP)-induced AKI in rats *via* miR-27a-3p/TAB3 axis, presenting in reducing kidney injury, ameliorated renal function, inflammation, and cell death (Wang et al., 2020a). Moreover, downregulation of NEAT1 ameliorated LPS-induced inflammatory responses by promoting macrophage M2 polarization *via* the miR-125a-5p/TRAF6/TAK1 axis (Wang and Guo, 2020).

Another lncRNA plasmacytoma variant translocation 1 (PVT1) has also gained much attention. Suppression of PVT1 in LPS-treated HK-2 cells could inactivate the NF-κB pathway *via* miR-17-5p, accompanied by improved cell viability and reduced inflammatory response (Yuan et al., 2021b). Besides, the reduction of PVT1 is also the mechanism of how curcumin protects against septic kidney injury (Huang et al., 2020). Additionally, PVT1 knockdown inhibited LPS-induced cell pyroptosis targeting the miR-20a-5p/NLRP3 signaling pathway (Deng et al., 2021b). The lncRNA DLX6 antisense RNA1 (DLX6-AS1) and the function of lncRNA maternally expressed gene 3 (MEG3) could function as the enablers of cell pyroptosis in LPS-induced AKI by decreasing miR-223-3p and targeting MEG3/miR-21/PDCD4 axis and miR-18a-3p/GSDMD, respectively (Yang et al., 2018; Tan et al., 2020; Deng et al., 2021a).

Similar to the regulatory network between lncRNAs and miRNAs mentioned above, the connecting as follows could also play a role in promoting cell apoptosis or inflammation response in septic AKI by sponging the related miRNA, TapSAKI/miR-22/PTEN/TLR4/NF-κB (Shen et al., 2019), TapSAKI/miR-205/IRF3axis (Han et al., 2021b), SNHG14/miR-93 (Shi et al., 2021), SNHG14/miR-4953p/HIPK1 (Yang et al., 2021a), SNHG5/miR-374a-3p/TLR4/NF-κB (Wang et al., 2021), MIAT/miR-29a (Zhang et al., 2019), ANRIL/miR-199a (Zhu et al., 2020b), TCONS_00016233/miR-22-3p (Zhang et al., 2020b), SIKIAT1/miR-96-3p/FOXA (Lu et al., 2020b), RMRP/miR-206 (Zhang et al., 2021), NKILA/miR-140-5p/CLDN2 (Han et al., 2021a), and XIST/miR-15a-5p/CUL3 (Xu et al., 2019).

Genes that expression elevated in AKI always play a role in promoting disease progression. Correspondingly, genes down-regulated exhibited the potential to suppress disease progression. There are, of course, exceptions. In the studies of podocyte injury of AKI, lncRNA growth arrest-specific transcript 5 (GAS5) expression decreased in a time-dependent manner, and GAS5 inhibition promoted podocyte injury by inhibiting the expression of PTEN *via* mediating the PI3K/AKT pathway (Fang et al., 2018).

### LncRNAs Exerted Nephroprotective Effects on Septic AKI

Contrary to the abovementioned studies, many expressions decreased lncRNAs could also participate in critical pathophysiological processes in septic AKI. PlncRNA-1 was downregulated in the serum of patients with septic AKI and LPS-induced cells. PlncRNA-1 overexpression relived kidney injury by increasing proliferation and inhibiting apoptosis and autophagy in LPS-treated cells (Fu et al., 2018). LncRNA differentiation antagonizing non-protein coding RNA (DANCR) was also decreased in the serum of AKI patients and LPS-treated HK-2 cells. This study demonstrates that septic AKI development could be alleviated by overexpressing DANCR, which comes with the miR-214 and Krüppel-like factor 6 expression declined (Zhao et al., 2020).

Overexpressing lncRNA6406 also attenuates LPS-stimulated AKI by mitigating cell inflammation, oxidative stress, and apoptosis *via* modulating miR-687/PTEN signaling (Liu et al., 2020). Additionally, lncRNA HOXA-AS2 exhibited protection in sepsis-engendered AKI by targeting miR-106b-5p and hindering the Wnt/β-catenin and NF-κB pathways (Wu et al., 2020b). Similarly, long intergenic non-protein coding RNA261(LINC00261) could function as a sponge to combine with microRNA-654-5p, which inhibits NF-κB activity by targeting the suppressor of cytokine signaling 3 (Li et al., 2021c). Long noncoding RNA cancer susceptibility candidate 2 (CASC2) has also been shown to protect against sepsis induced AKI by blocking the miR155 and NF-κB signaling pathways (Wang et al., 2020d). Furthermore, CASC2 overexpression facilitated cell viability and restrained cell apoptosis, migration, epithelial-mesenchymal transition (EMT), and oxidative stress through regulating the miR-545-3p/PPARA axis in LPS-triggered HK-2 and HEK293 cells (Hu et al., 2021).

LncRNA Colon cancer-associated transcript-1 (CCAT1) was shown to have anti-inflammation and pro-survival effects in LPS induced renal tubular epithelial cell damage *in vitro* and *in vivo*. This study also revealed the mechanism of dysregulated CCAT1/miR-155/SIRT1 pathway regulated disease progression in a murine model of LPS-induced AKI (Lu et al., 2020a). A recent study identified LncRNA NONRATG019935.2 exhibited a significant reduction both in septic AKI rats and LPS-treated NRK-52E cells. Similarly, overexpression of NONRATG019935.2 suppressed cell apoptosis and p53 protein levels in LPS-treated NRK-52E cells and retarded septic AKI development in the rat model of septic AKI (Ding et al., 2021).

### LncRNAs Showed a Bidirectional Effect on Septic AKI

Several lncRNAs also showed a bidirectional effect on septic AKI in the current study. For example, LPS treatment increased ncRNA colorectal neoplasia differentially expressed gene (CRNDE) expression in HK-2 cells and mice. CRNDE inhibition could reduce sepsis-induced kidney injury by blocking the activation of the TLR3/NF-κB pathway (Sun et al., 2019). Meanwhile, overexpressing CRNDE activated TLR4/NF-κB signaling by regulating miR-146a, which accelerated LPS-induced inflammation and apoptosis in HK-2 cells (Wu et al., 2020a). In contrast, the opposite results have been found in the urine-derived septic rats, and in LPS-treated HK-2 and HEK293 cells, CRNDE was markedly down-regulated. Down-regulation of CRNDE could aggravate kidney injury *via* increasing miR-181a-5p (Wang et al., 2020c). Another lncRNA with a dual effect in septic AKI is lncRNA HOX transcript antisense RNA (HOTAIR). Overexpression of HOTAIR inhibited apoptosis by downregulating miR-34a/Bcl-2 signaling in the cecal ligation puncture septic AKI rats (Jiang et al., 2019b). However, in the urine-derived sepsis rat model, HOTAIR was up-regulated, and it promoted HK-2 cell apoptosis through the miR-22/HMGB1 pathway (Shen et al., 2018).

## LncRNAs in I/R Induced AKI

### LncRNAs Changed Globally in I/R Induced AKI

Ischemia–reperfusion is one of the leading causes of AKI. Inflammation and oxidative stress are the most common processes involved in the pathogenesis of renal I/R injury. Zhou and coworkers profiled the lncRNA expression pattern in the kidney of AKI mice by second-generation gene sequencing. They reported 90 differentially expressed lncRNAs which included 20 up-regulated and 70 down-regulated. These dysregulated lncRNAs participated in multiple biological processes, including stimulus response, multicellular organismal processes, single-multicellular organism processes, and so on (Zhou et al., 2017a). In 2017, a study reported 52 differentially expressed lncRNAs in the kidney, where three significantly up-regulated lncRNAs (TCONS_00042175, TCONS_00058568, and TCONS_00047728) were negatively correlated with I/R-induced kidney injury (Liu et al., 2017). Another study revealed the altered ncRNA in I/R-induced AKI, which covered the change of lncRNAs and showed the lncRNA-miRNA-mRNA regulatory network based on ceRNA theory. In a recent study, Tao et al. also reported that a total of 2,267 lncRNAs were prominently expressed in experimental I/R mice (Tao et al., 2019).

### LncRNAs Promoting I/R Induced AKI

Studies have been conducted to determine if lncRNAs are implicated in I/R-induced AKI (Table 2). Hypoxia contributes to renal functional decline during I/R injury. LncRNA MALAT1 was activated by hypoxia-inducible factor 1-α (HIF-1α) and negatively regulated the expression of IL-6, TNF-α, and NF-kB (Kölling et al., 2018). Another HIF-1α dependent lncRNAs psoriasis susceptibility-related RNA gene induced by stress (Prins) has been shown to increase in I/R damaged kidney and interact with RANTES to mediate various inflammatory responses (Yu et al., 2016).

**Table 2:** LncRNAs in I/R-induced AKI.

AKI	lncRNA	Expression	Regulation network	Function	Reference
I/R-AKI	MALAT1	up	NF-κB	Inhibit the hypoxia-induced inflammatory response	Kölling et al. (2018)
	PRINS	up	RANTES	Promote inflammation	Yu et al. (2016)
	SNHG14	up	miR-124-3p/ MMP2	Promote inflammation and oxidative stress	Xue et al. (2021)
	EGOT	down	HuR-ATG7/16 L1	Promote the hypoxia-induced autophagy	Wang et al. (2020b)
	XIST	up	miR-124-3p/ITGB1	Promote apoptosis and inflammation	Chen et al. (2020)
			miR-142-5p/ PDCD4	Promote CoCl2-induced cellular apoptosis	Tang et al. (2020)
	NEAT1	up	miR-27a-3p	Promote CoCl2-induced cellular apoptosis	Jiang et al. (2019a)
	GAS5	up	miR-21	Promote cell apoptosis	Geng et al. (2020)
	LINC00520	up	miR-27b-3p/OSMR	Promote cell apoptosis	Tian et al. (2019)
	LINC00963	up	miR-128-3p/ JAK2/STAT1	Reduce G1 arrest and apoptosis	Xie et al. (2020)
	MEG3	up	miR-145-5p /Wnt/β-catenin	Activate mitophagy and induce apoptosis	Liu et al. (2021)
	LINC00052	down	miR-532-3p/ Wnt/β-catenin	Decrease hypoxia-induced ROS and MDA accumulation	Li et al. (2020)
	H19	up	miR-130a/BCL2L11	Augment cell apoptosis	Yuan et al. (2021a)
			miR-30a-5p	Reduce cell apoptosis and inflammation	Haddad et al. (2021)
	TUG1	down	miR-494-3p/ E-cadherin	Alleviate cell apoptosis	Chen et al. (2021b)

*In vitro* experiments, the lncRNA SNHG14 could mitigate I/R-induced kidney injury *via* competition with miR-124-3p to regulate the expression of MMP2 in HK-2 cells (Xue et al., 2021). The decline of lncRNA eosinophil granule ontogeny transcript (EGOT) promoted hypoxia-induced autophagy in HK-2 cells *via* binding with the RNA-binding protein Hu antigen R (HuR) and regulation of ATG7/16 L1 expression (Wang et al., 2020b). The other three expressions increased lncRNA X-inactive specific transcript (XIST), NEAT1, and GAS5 also promoted cell apoptosis and inflammation through the modulation of miR-124-3p, miR-27a-3p, and miR-21, separately (Jiang et al., 2019a; Chen et al., 2020; Geng et al., 2020). Meanwhile, other researchers have experimentally validated the upregulation of lncRNA XIST in I/R-AKI and showed its regulatory role of miR-142-5p and PDCD4 (Tang et al., 2020). Signaling pathway analysis was performed by mapping AKI related lncRNAs to KEGG pathways. One study reported that lncRNA LINC00520/miR-27b-3p/OSMR axis contributed to the AKI aggravation by activating the PI3K/AKT pathway (Tian et al., 2019). LINC00963, another lncRNA interplay with miR-128-3p, could promote AKI through the JAK2/STAT1 pathway (Xie et al., 2020). The lncRNA MEG3 has also been found to trigger the Wnt/β-catenin pathway to aggravate kidney I/R injury (Liu et al., 2021).

### LncRNAs Exerted Nephroprotective Effects on I/R-AKI

So far, only one lncRNA has been found to have a protective effect on I/R-AKI. The lncRNA LINC00052 expression was significantly decreased in AKI patient plasma but enormously elevated in hypoxic cells. Overexpression of LINC00052 could ameliorate AKI by reactivating Wnt/β-catenin signaling, which is inactivated in NRK-52E cells under hypoxic conditions *via* sponging miR-532-3p (Li et al., 2020).

### LncRNAs Showed a Bidirectional Effect on I/R-AKI

LncRNA H19 and lncRNA TUG1 harbored a bidirectional regulation of cellular function in IR-induced AKI. The lncRNA H19 could regulate the miR-130a/BCL2L11 axis to modulate the proliferation and apoptosis of HK-2 cells under hypoxia/reoxygenation conditions. In parallel, downregulation of lncRNA H19 could promote cell proliferation, inhibit cell apoptosis, and suppress inflammatory cytokine expression in HK-2 cells throughout the miR-130a/BCL2L11 pathway (Yuan et al., 2021a). However, another study has found a contrary function of H19 in I/R-AKI. George et al. pointed out that overexpression of lncRNA H19 in the I/R model could provide a significant renal-protective effect through sponging of microRNA-30a-5p (Haddad et al., 2021). Overexpressing lncRNA TUG1 diminished the protective effect of total glucosides of paeony on AKI and exacerbated autophagy in HK-2 cells (Chang et al., 2021b). However, Chen et al. found that overexpression of lncRNA TUG1 could significantly alleviate cell apoptosis by serving as a miR-494-3p sponge to disinhibit E-cadherin (Chen et al., 2021b).

## LncRNAs in Post-Contrast AKI

Post-contrast acute kidney injury (PC-AKI), also called contrast-induced acute kidney injury (CI-AKI), is a kind of kidney disease that the renal function deteriorates suddenly within 48 h of intravascular administration of contrast medium (van der Molen et al., 2018). Data have shown that PC-AKI was the third most common cause of AKI in hospitalized patients (Nash et al., 2002) and accounted for high mortality rates (Levy et al., 1996). More recently, the literature has emerged that offers significant findings of the definition, risk factors, pathophysiology mechanism, and medical intervention of PC-AKI (Mehran et al., 2019; Bansal and Patel, 2020; Everson et al., 2020). Bioinformatics analysis revealed that lncRNAs could serve as novel biomarkers in the early phase of PC-AKI (Table 3). Chen et al. depicted the expression landscape of lncRNAs and identified 357 differentially expressed lncRNAs in PC-AKI. Among these, lnc-HILPDA and lnc-PRND, which were conservative and remarkably up-regulated in both kidneys and blood from rats and the blood of PC-AKI patients, can effectively predict PC-AKI risk and precisely distinguish PC-AKI patients after the exposure to contrast medium (Chen et al., 2021a).

**Table 3:** LncRNAs in PC-AKI, post-transplant AKI, and cisplatin induced AKI.

AKI	LncRNA	Expression	Regulation network	Function	Reference
PC-AKI	LNC_000343	down	rno-miR-1956-5p/KCP	Reduce interstitial fibrosis and renal injury	Cheng et al. (2019)
	NONRATT025462.2	down	rno-miR-126a-5p, miR-200a-5p, miR-200a-5p/Cndp1	Associated with the process of antioxidation	Bao et al. (2021)
	NONRATT020679.2	down	rno-miR-126a-5p, miR-200a-5p/Tmem184b	-	Bao et al. (2021)
Post-transplant AKI	ATB	up	miR-200c /TGF-β	Activate cell proliferation and cyclosporine A-mediated apoptosis	Qiu et al. (2017)
	XIST	-	miR-212-3p/ ASF1A, BRWD1	Regulate inflammatory and apoptosis	Cheng and Wang (2020)
			miR-122-5p/ PFKFB2	Influence thyroid hormone and AMPK signaling	Cheng and Wang (2020)
Cisplatin induced AKI	LncRNA 9,884	up	NF-κB/macrophage migration inhibitory factor	Aggravate tubular epithelial cells injury	Zhang et al. (2020a)
	MEG3	up	AKT/TSC/mTOR	Promote cisplatin-induced nephrotoxicity	Jing et al. (2021)
	GAS5	up	miR-205-5p	Aggravate renal epithelial cell apoptosis	Zhang et al. (2021)
	OIP5-AS1	down	miR-144-5p/PKM2	Reduce the apoptosis of renal epithelial cells	Chang et al. (2021a)
	XLOC_032768	down	TNF-α	Reduce cell apoptosis	Zhou et al. (2020)
	PRNCR1	down	miR-182-5p/EZH1	Reduce cell apoptosis	Li et al. (2021a)

Since the expression of lncRNAs has undergone massive changes in PC-AKI, more and more researchers are delving into the specific regulatory mechanism that lncRNA is involved in PC-AKI. Cheng and coworkers systematically explored the lncRNA-associated-ceRNA regulatory network in rats with PC-AKI by RNA-seq analysis and two constructed ceRNA regulatory pathways in the CI-AKI rat model (novel_circ_0004153/rnomiR-144-3p/Gpnmb or Naglu and LNC_000343/rno-miR-1956-5p/KCP) were reported firstly and validated by real-time qPCR. Further function annotation shows that the disease-specific lncRNAs may participate in physiological and pathological processes of PC-AKI from different aspects, including porphyrin-containing compound metabolic, biosynthetic processes, heme binding, and so on (Cheng et al., 2019). Another study also found and verified that differential expressed lncRNAs and their co-expression targets were associated with vital pathological pathways of PC-AKI, like inflammation and oxidative stress (Bao et al., 2021). For instance, they identified a group of lncRNAs (such as MSTRG.11448.4, MSTRG.2420.1, MSTRG.6245.1, and NONRATT023367.2) linked to glycoprotein non-metastatic melanoma protein, which can negatively regulate inflammation in AKI. Furthermore, lncRNA-associated ceRNA analysis revealed a Cndp1-specific network that was also likely to be related to the process of antioxidation, which plays a vital role in oxidative stress-mediated injury in PC-AKI (Ripoll et al., 2007; Zhou et al., 2017b). In summary, although hundreds of lncRNAs associated explicitly with the PC-AKI have been identified and several studies have constructed their regulatory networks with other RNAs, the specific biological functions and detailed mechanisms of the vast majority of lncRNAs remain unclear, and further research in this area is required.

## LncRNA in Kidney Transplant Acute Kidney Injury

Kidney transplantation is one of the effective treatments for patients with end-stage renal disease (ESRD). However, as a potentially severe complication, post-transplant AKI still maintains high morbidity and mortality rates (Mittal and Kohli, 2014). The pathogenesis of post-transplant AKI is complex. Several researchers have found the association between lncRNA and post-transplant AKI (Table 3).

In 2013, Sui et al. integrated protein array-based proteomics and RNA microarray-based genomics to investigate the transcription factors (TF), miRNAs, long noncoding RNA of biopsies of three patients with acute rejections. They discovered that among the 32 differentially expressed lncRNAs, FXYD1 and HOXA11 were targeted individually by the lncRNAs Uc002nyb and Uc003syy, which were linked to a poor outcome in acute rejections (Sui et al., 2013). Another study also used lncRNA microarrays to compare the lncRNA expression in kidney biopsy from acute rejection following kidney transplantation. After validating the microarray results by qRT-PCR and screening in the five databases, the functions of five candidate lncRNAs (AF113674, uc003wbj, uc010ftb, uc001fty, and AK129917) were further defined by their location to the gene on the genome (Chen et al., 2014). Other studies have investigated the correlation between post-transplant AKI-specific lncRNA expression and the diagnosis or prognosis of acute rejection. LncRNA AF264622 and AB209021 exhibited excellent diagnostic performance both in pediatric and adult renal transplants (Ge et al., 2017). Furthermore, lncRNA MIR155HG, which participates in allograft rejection associated pathways, such as graft versus host disease, T-cell and B cell receptor, signaling pathways, can predict the risk of graft loss effectively (Zou et al., 2019).

Notably, lncRNAs are also released and circulated in the plasma, urine, and other body fluids. Investigators have also found lncRNAs in blood or urine that could be used for early detection of post-transplant AKI (Lorenzen et al., 2015; Ge et al., 2017). However, studies describing the mechanism of lncRNA in post-transplant AKI are limited. LncRNA-ATB, a novel lncRNA activated by TGF-β, may contribute to kidney transplantation survival through mediating the TGF-β signaling pathway. In this study, the expression of lncRNA-ATB was largely up-regulated in the TGF-β treated human tubule cells, activating cell proliferation and cyclosporine A-mediated apoptosis of renal cells *via* the downregulation of miR-200c (Qiu et al., 2017). Another up-regulated lncRNA in the serum of a post-transplant patient with AKI is XIST which could serve as a ceRNA to sponge hsa-miR-212-3p to regulate inflammation and apoptosis in the progression of AKI *via* modulating the expression of ASF1A and BRWD1. Furthermore, lncRNA XIST could also sponge miR-122-5p to influence thyroid hormone and AMPK signaling *via* modulating the expression of PFKFB2 in AKI (Cheng and Wang, 2020).

Overall, these studies reasonably provide consistent evidence of an association between lncRNA and post-transplant AKI. Most current research on lncRNA remains at the level of their expression pattern identification and diagnostic or prognostic potential in diseases. There is still much room for improvement, and more attention should be paid to their therapeutic potential.

## LncRNA of AKI Induced by Other Causes

Aside from the four types of AKI mentioned above, medication or toxin-induced AKI is a kind of AKI that is common in the clinic but poorly understood in lncRNA-based biological mechanism research. Upon closer inspection of the literature， We identified only six relevant papers investigating the role of lncRNA in cisplatin reduced AKI. At the same time, no study reports the relationship between lncRNAs in other common drug-induced AKI, such as aristolochic acid, folic acid, and antibiotics for the time being (Table 3). Among them, lncRNA9884 is a promoting factor of cisplatin induced AKI. Expression elevated lncRNA9884 could significantly aggravate tubular epithelial cells injury by activating inflammatory signaling pathway NF-κB/macrophage migration inhibitory factor (Zhang et al., 2020a). Another recent study has described that the lncRNA MEG3 could promote cisplatin-induced nephrotoxicity through regulating AKT/TSC/mTOR-mediated autophagy, and LncRNA GAS5 could aggravate renal epithelial cell apoptosis in cisplatin-induced AKI by regulating miR-205-5p (Jing et al., 2021; Zhang et al., 2021).

One study found that lncRNA OIP5-AS1 was significantly downregulated in cisplatin-induced AKI model both *in vitro* and *in vivo*. Further research revealed that OIP5-AS1 could reduce the apoptosis of cisplatin-stimulated renal epithelial cells to exert a protective effect by targeting the miR-144-5p/PKM2 axis (Chang et al., 2021a). Similarly, the novel lncRNA XLOC_032768 and PRNCR1 could also perform a similar role of reducing cisplatin-induced apoptosis of renal tubular epithelial cells to protect against the cisplatin-induced AKI through TNF-α and miR-182-5p/EZH1, respectively (Zhou et al., 2020; Li et al., 2021a). Regrettably, as for the other reasons induced AKI, for instance, rhabdomyolysis-associated AKI and trauma associated AKI, the biological mechanism of those diseases has been studied, but not for the role of lncRNAs.

## Discussion

**Figure 2:**
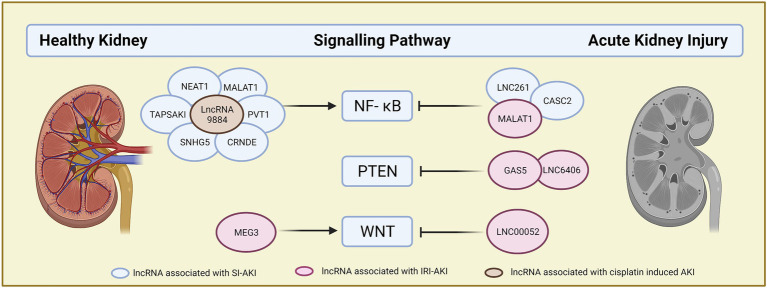
Canonical lncRNAs signaling pathway. LncRNAs are summarized by their function in key signaling pathways in AKI. LncRNAs on the left are the lncRNA which could activate the signaling pathway, while lncRNAs on the right are the lncRNA which could inactivate the signaling pathway. Different colors of circles indicate the different types of AKI. Blue circle: sepsis induced AKI, red circle: ischemic/perfusion induced AKI, brown circle: cisplatin induced AKI.

LncRNAs offer a novel direction for pathophysiological study and the search for new therapeutic targets in acute kidney injury. On the one hand, lncRNAs exhibit the potential to act as biomarkers for the early diagnosis and prognosis of patients with AKI. On the other hand, lncRNAs have been identified as the key mediators that participated in the pathophysiology of AKI caused by different reasons. Expression, function, and regulation network of lncRNAs altered depending on the causes of AKI. Several identified lncRNAs, such as XIST, MALAT1, NEAT1, and so on, contribute to kidney injury in two or more different reasons caused AKI through different regulatory mechanisms. Those lncRNAs may represent the potential therapeutic target for the AKI. Besides, based on the ceRNA regulatory network, one lncRNA can be involved in the different signaling pathways and link with different targets downstream, which are closely related to the process of cell apoptosis, inflammation, such as NF-κB, WNT, and PTEN (Figure 2). This feature greatly enriched and complicated the regulatory mechanism of lncRNA and provided a way to develop new therapeutic solutions for AKI.

Although the function and the diagnostic and prognostic roles of lncRNAs in AKI had been carried out extensively, the limitations are also evident in the current study. First, most studies of lncRNA function were based on the ceRNA network, which could only reveal lncRNA function at the post-translational level, possibly because of the use of bioinformatics to predict the functions of lncRNAs is preferred over traditional, time-consuming, and expensive experimental methods. Neither the function of lncRNAs at the epigenetic level or transcription level, nor the cell organelle-specific function of lncRNAs gained equitable attention in lncRNAs in AKI. Second, the specific mechanisms of various studies are still unclear, and more in-depth research is needed to reveal the relevant mechanisms. Finally, clinical trials to validate the therapeutic potential of lncRNAs in AKI are currently inadequate due to the lack of a safe and effective delivery mechanism. There is still a long way to go before the study results can be promoted clinically. For sure, some difficulties exist in the lncRNAs research due to the low expression, low sequence conservation, and diverse secondary functional structure of the lncRNAs. Thus, in the future, the more credible algorithms need to be developed, the more precise detection technology of lncRNAs needs to be innovated, the more safe and efficient drug delivery method needs to be devised. In summary, there remains a huge gap in access to a comprehensive understanding of LncRNAs in AKI, and further efforts will be needed.

## Author Contributions

LY and BW defined the subject, revised the literature, and wrote the manuscript. All authors contributed to the article and approved the submitted version.

## Funding

This study was supported by the National Key R&D Program of China (2020YFC2005000). Figures 1 and 2 were created with a free version of Biorender.com

## Conflict of Interest

The authors declare that the research was conducted in the absence of any commercial or financial relationships that could be construed as a potential conflict of interest.

## Publisher’s Note

All claims expressed in this article are solely those of the authors and do not necessarily represent those of their affiliated organizations, or those of the publisher, the editors and the reviewers. Any product that may be evaluated in this article, or claim that may be made by its manufacturer, is not guaranteed or endorsed by the publisher.
