# Supplementary material for: An Update of Long-Noncoding RNAs in Acute Kidney Injury
Source: Front Physiol. 2022 Mar 8;13:849403. doi: 10.3389/fphys.2022.849403 (PMC8957988; doi:10.3389/fphys.2022.849403)
Supplement: Supplementary file 1 [file Table_1.DOCX]

Table 1. LncRNAs in septic AKI.

| AKI | lncRNA | Expression | Regulation Network | Function | Ref |
| --- | --- | --- | --- | --- | --- |
| Septic AKI | MALAT1 | up | miR-146a/NF-κB | Promote cytokine creation and immune response | (25) |
|  | NEAT1 | up | miR-22-3p/NF-κB | Promote cell apoptosis | (28) |
|  |  |  | let-7b-5p/TRAF6 | Promote inflammation | (29) |
|  |  |  | miR-93-5p/TXNIP | Promote apoptosis, inflammation, and oxidative stress | (30) |
|  |  |  | miR-27a-3p/TAB3 | Promote cell apoptosis | (31) |
|  |  |  | miR-125a-5P/TRAF6/TAK1 | Modulate macrophage polarization | (32) |
|  | PVT1 | up | miR-17-5p/ NF-κB | Promote cell apoptosis | (33) |
|  |  |  | miR-20a-5p/NLRP3 | Promote cell pyroptosis and inflammatory factors creation | (35) |
|  | DLX6-AS1 | up | miR-223-3p/NLRP3 | Improve cytotoxicity | (36) |
|  | MEG3 | up | miR-21/PDCD4 | Promote cell apoptosis | (37) |
|  |  |  | miR-18a-3p/GSDMD | Promote pyroptosis | (38) |
|  | TapSAKI | up | miR-22/PTEN/TLR4/NF-κB | Promote cell apoptosis and inflammation response | (39) |
|  |  |  | miR-205/IRF3 | Enhance cytotoxicity | (40) |
|  | SNHG14 | up | miR-93/IRAK4/NF-κB | Accelerate cellular injury | (41) |
|  |  |  | miR-495-3p/HIPK1 | Inhibit cell proliferation and autophagy | (42) |
|  | SNHG5 | up | miR-374a-3p/TLR4/NF-κB | Promote cell apoptosis | (43) |
|  | MIAT | up | miR-29a | Promote cell apoptosis | (44) |
|  | ANRIL | up | miR-199a/TLR4/NF-kB | Promote cell apoptosis | (45) |
|  | TCONS_00016233 | up | miR-22-3p/AIFM1 | Promote LPS-induced HK-2 cell apoptosis and the expression of IL-1b and TNF-a | (46) |
|  | SIKIAT1 | up | miR-96-3p | Promote cell apoptosis | (47) |
|  | RMRP | up | miR-206/DPX5 | Promote cell apoptosis | (48) |
|  | NKILA | up | miR-140-5p/CLDMA | Promote cell apoptosis | (49) |
|  | XIST | up | miR-15a-5p/ CUL3 | Enhance podocyte cell apoptosis | (50) |
|  | GAS5 | down | PI3K/AKT | Promotes podocyte injury | (51) |
|  | PlncRNA-1 | down | - | Promote cell proliferation, inhibit apoptosis and autophagy | (52) |

Table 1. LncRNAs in septic AKI (continued).

| AKI | lncRNA | Expression | Regulation Network | Function | Ref |
| --- | --- | --- | --- | --- | --- |
| Septic AKI | DANCR | down | miR-214/KLF6 | Suppress cell apoptosis and cytokine creation | (53) |
|  | lncRNA6406 | down | miR-687/PTEN | Alleviate inflammation, oxidative stress, and inhibit apoptosis | (54) |
|  | HOXA-AS2 | down | miR-106b-5p/Wnt/β-catenin/ NF-κB | Inhibit the inflammation | (55) |
|  | LINC00261 | down | miR-654-5p/SOCS3/NF-κB | Improve cell viability, suppress the apoptosis, and reduced the generation of inflammation cytokines | (56) |
|  | CASC2 | down | miR-155/NF-κB | Inhibit inflammation factors creation, cell apoptosis and oxidative stress | (57) |
|  |  |  | miR-545-3p/PPARA | Facilities cell viability and restrain cell apoptosis migration EMT and oxidative stress | (58) |
|  | CCAT-1 | down | miR-155/SIRT1 | Attenuate inflammatory response and apoptosis | (59) |
|  | NONRATG019935.2 | down | P53 | Suppress the apoptosis | (60) |
|  | CRNDE | up | TLR3/NF-κB | Promote kidney injury | (61) |
|  |  |  | miR-146a /TLR4/NF-κB | Accelerate LPS-induced inflammation and apoptosis | (62) |
|  |  | down | miR-181a-5p | Accelerate LPS-induced inflammation and apoptosis | (63) |
|  | HOTAIR | up | miR-34a/Bcl-2 | Inhibit the apoptosis of kidney tissues | (64) |
|  |  |  | miR-22/HMGB1 | Promote HK-2 cell apoptosis | (65) |
